# Inhibition of inducible Nitric Oxide Synthase by a mustard gas analog in murine macrophages

**DOI:** 10.1186/1471-2121-7-39

**Published:** 2006-11-30

**Authors:** Min Qui, Victor M Paromov, Hongsong Yang, Milton Smith, William L Stone

**Affiliations:** 1 Department of Pediatrics, East Tennessee State University, Johnson City, TN, USA; 2 Amox Ltd., Lawton, MI 49605, USA

## Abstract

**Background:**

2-Chloroethyl ethyl sulphide (CEES) is a sulphur vesicating agent and an analogue of the chemical warfare agent 2,2'-dichlorodiethyl sulphide, or sulphur mustard gas (HD). Both CEES and HD are alkylating agents that influence cellular thiols and are highly toxic. In a previous publication, we reported that lipopolysaccharide (LPS) enhances the cytotoxicity of CEES in murine RAW264.7 macrophages. In the present investigation, we studied the influence of CEES on nitric oxide (NO) production in LPS stimulated RAW264.7 cells since NO signalling affects inflammation, cell death, and wound healing. Murine macrophages stimulated with LPS produce NO almost exclusively via inducible nitric oxide synthase (iNOS) activity. We suggest that the influence of CEES or HD on the cellular production of NO could play an important role in the pathophysiological responses of tissues to these toxicants. In particular, it is known that macrophage generated NO synthesised by iNOS plays a critical role in wound healing.

**Results:**

We initially confirmed that in LPS stimulated RAW264.7 macrophages NO is exclusively generated by the iNOS form of nitric oxide synthase. CEES treatment inhibited the synthesis of NO (after 24 hours) in viable LPS-stimulated RAW264.7 macrophages as measured by either nitrite secretion into the culture medium or the intracellular conversion of 4,5-diaminofluorescein diacetate (DAF-2DA) or dichlorofluorescin diacetate (DCFH-DA). Western blots showed that CEES transiently decreased the expression of iNOS protein; however, treatment of active iNOS with CEES *in vitro *did not inhibit its enzymatic activity

**Conclusion:**

CEES inhibits NO production in LPS stimulated macrophages by decreasing iNOS protein expression. Decreased iNOS expression is likely the result of CEES induced alteration in the nuclear factor kappa B (NF-κB) signalling pathway. Since NO can act as an antioxidant, the CEES induced down-regulation of iNOS in LPS-stimulated macrophages could elevate oxidative stress. Since macrophage generated NO is known to play a key role in cutaneous wound healing, it is possible that this work has physiological relevance with respect to the healing of HD induced skin blisters.

## Background

HD is a chemical weapon that can produce casualties in military situations and has been used with devastating results against civilian populations [1]. Extensive and slow healing lesions following exposure to HD can place a heavy burden on the medical services of military and public health organizations. The design of effective countermeasures to HD depends upon a detailed understanding of the molecular mechanisms for its toxicity. Important mechanisms of HD induced skin injury are alkylation of DNA and other macromolecules, accompanied by enhanced reactive oxygen species (ROS) generation and depletion of intracellular glutathione (GSH) [2-5]. Depletion of GSH by HD and its metabolites is known to shift the intracellular redox milieu toward a more oxidized state with a subsequent loss of protection against oxidative free radicals and an activation of inflammatory responses[6,7].

It has been shown that HD induces a vast "spectrum" of inflammatory cytokines released from keratinocytes [8,9]. It is likely that CEES cause similar changes in macrophages and leukocytes. We previously found that LPS, as well as inflammatory cytokines, such as tumor necrosis factor-alpha (TNF-α) and interleukin one-beta (IL-1β), significantly amplify the toxicity of CEES in RAW264.7 macrophages [10]. In macrophages, stimulation by LPS, as well as by pro-inflammatory cytokines, leads to the activation and nuclear translocation of NF-κB [11]. One of the major consequences of such activation in macrophages is an induction of iNOS expression with subsequent elevation of intracellular NO [12]. The effect of CEES on NO generation and on the NF-κB pathway is potentially significant since NO signalling plays an important role in inflammation, the mechanisms of cell death NF-κB [13,14], and wound healing [15,16]. The present work describes the inhibition of NO production and iNOS expression in LPS stimulated macrophages treated with CEES.

## Results

### CEES transiently suppresses NO production and iNOS expression in LPS stimulated cells

**Figure 1:**
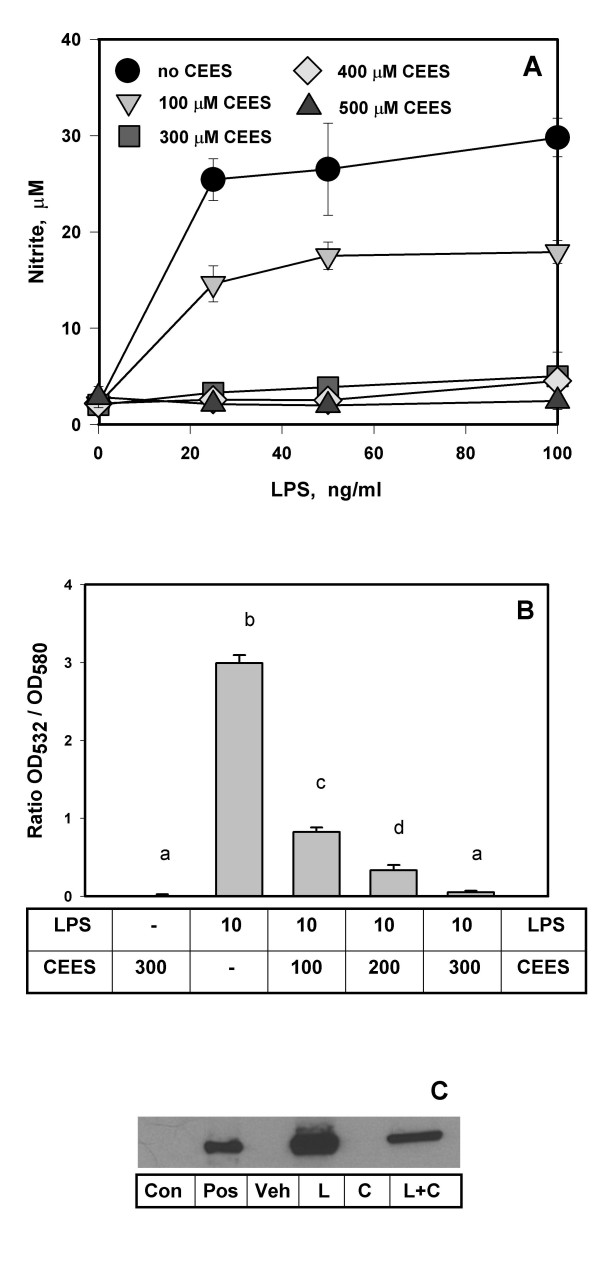
**CEES inhibits NO production and iNOS expression in LPS stimulated RAW264.7 macrophages**. *Panel A*: Macrophages were simultaneously treated with various levels of CEES (as indicated) and low doses of LPS (as indicated). NO production was monitored as the concentration of nitrite in the culture medium after 24 h. *Panel B*: Cells were treated similarly as for *Panel A*; LPS, 10 ng/ml; CEES, 100, 200, or 300 μM (as indicated). Means not sharing a common letter are significantly different (p < 0.05). Nitrite levels in the culture medium (OD at 532 nm) were normalized to the amount of viable cells (OD of the MTT product at 580 nm). *Panel C*: Western blot analysis of iNOS protein from cells simultaneously incubated with 300 μM CEES and/or 10 ng/ml LPS for 24 h; cell lysates were prepared as described in Materials and Methods: Con, control cells; Pos, iNOS protein for positive control; Veh, vehicle; L, 10 ng/ml LPS stimulated cells; C, 300 μM CEES treated cells; L+C, LPS/CEES treated cells.

In Figure 1a, we examined nitrite secretion into the cell culture medium by RAW 264.7 murine macrophages after 24 hours of treatment with CEES and various levels of LPS. Nitrite level in the cell culture medium, as measured by the Griess reagent, is a reliable indicator of nitric oxide secretion. These data show that CEES (100–500 μM) inhibited the secretion of NO into the cell medium by LPS stimulated macrophages in a dose-dependent manner. Low levels of CEES (≤ 100 μM) only partially inhibited NO production, whereas levels higher than 300 μM completely inhibited NO production. Although CEES does decrease the viability of LPS stimulated macrophages [10], the decreased generation of NO cannot be accounted simply for the loss of viable cells. Figure 1b shows that in case nitrite levels in the culture medium (as measured by OD at 532 nm) are normalized to the amount of viable cells (OD at 580 nm, MTT assay, measured separately) there is still a significant CEES dose dependent inhibition of NO formation.

In order to determine if CEES influenced cellular levels of iNOS, we performed Western blot analyses (Figure 1c) of the cell lysates using highly selective anti-iNOS antibodies with equal amounts of total protein applied to each lane. Control RAW 264.7 macrophages had no detectable iNOS protein, CEES treatment alone did not induce any iNOS protein but LPS (10 ng/ml for 24 hours) produced a marked induction of iNOS protein. When simultaneously treated with LPS (10 ng/ml) and CEES (300 μM) there was a marked reduction in the LPS induction of iNOS protein.

**Figure 2:**
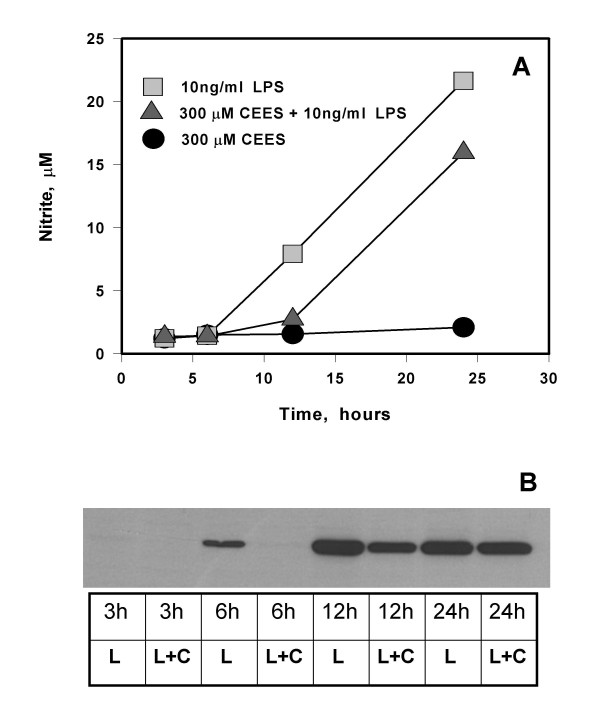
**Time course of NO production and iNOS expression in LPS stimulated RAW264.7 macrophages incubated with CEES**. *Panel A*: Macrophages were incubated with 10 ng/ml LPS alone, 300 μM CEES alone or simultaneously with both 300 μM CEES 10 ng/ml LPS for various time intervals (as indicated). NO production measured as concentration of nitrite in culture medium. *Panel B*: Western blot analysis of iNOS protein from the cells incubated with 300 μM CEES with or without 10 ng/ml LPS; cell lysates were prepared after 3, 6, 12, or 24 hour incubation (as indicated) as described in Materials and Methods; L, LPS; C, CEES.

We then examined the influence of 300 μM CEES on the time course of NO production in macrophage stimulated with 10 ng/ml LPS. Figure 2a shows that CEES delays, but does not prevent, the production of NO (as measured by nitrite formation) in LPS-stimulated macrophages. In fact, after 12 hours the rate of NO production is about the same in cells treated with LPS alone compared with cells treated with both LPS and CEES. Western blot data (Figure 2b) from the cells used in Figure 2a show a similar pattern: LPS alone induces robust iNOS protein expression which is completely inhibited by CEES for up to 6 hours. After 12 hours, however, the cells incubated with both CEES and LPS show a rebound in the expression of iNOS and after 24 hours the iNOS protein level in cells treated with both CEES and LPS is very similar to that observed in cells treated with LPS alone. These data show that the influence of CEES on both nitric oxide synthesis and iNOS expression is transient.

### CEES does not inhibit iNOS enzymatic activity *in vitro*

In order to evaluate the possible direct inhibitory effect of CEES on iNOS activity *in vitro*, we measured the intracellular rates of 4,5-diaminofluorescein (DAF-2) or dichlorofluorescin (DCFH) oxidation in intact macrophages. Dichlorofluorescin diacetate (DCFH-DA) is permeable to the cell plasma membrane and intracellular esterases convert it into a membrane impermeable (DCFH) form which is can be oxidized to highly fluorescent dichlorofluorescein (DCF) by free radicals. In macrophages, the oxidation of DCFH has been shown to be a sensitive and relatively selective probe for monitoring intracellular NO formation by iNOS [17].

**Figure 3:**
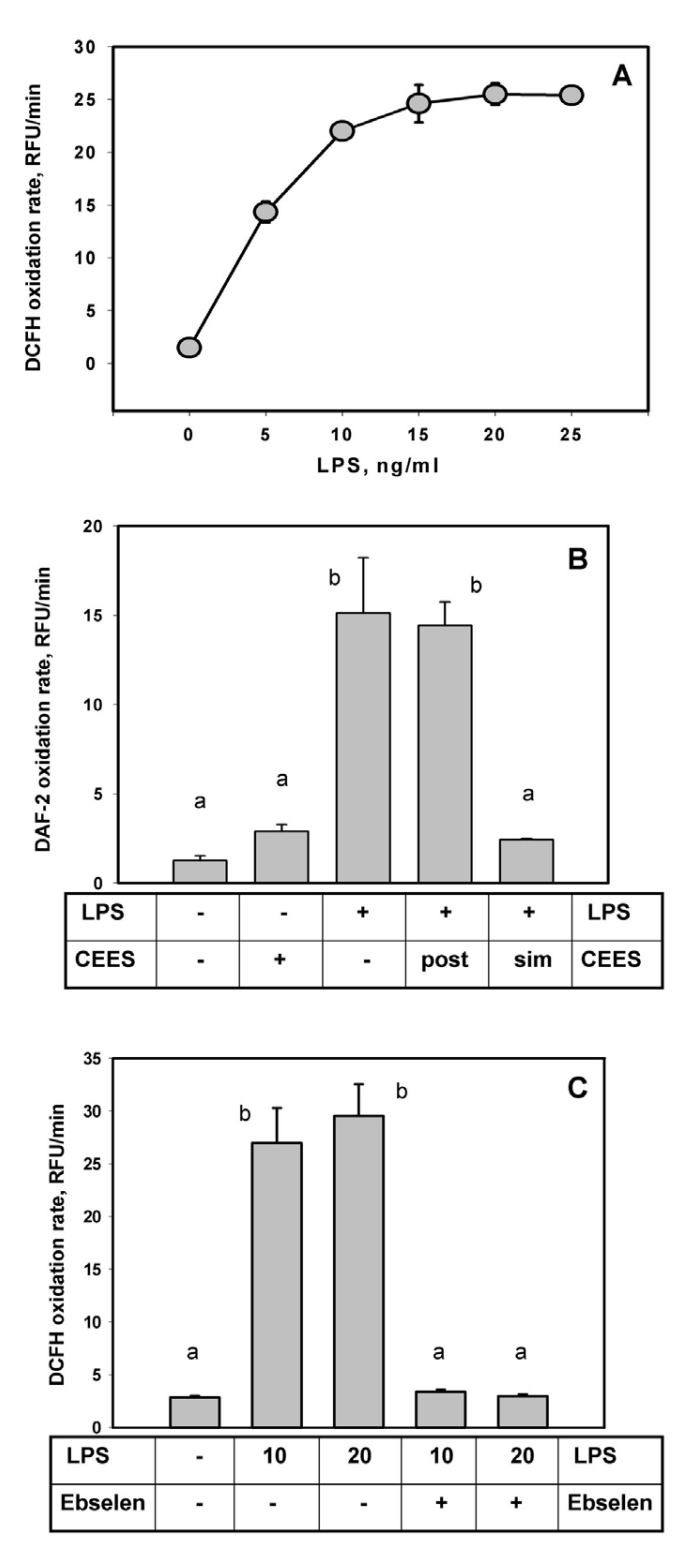
**CEES reduces intracellular NO in LPS stimulated RAW264.7 macrophages**. *Panel A*: Intracellular DCFH (20 μM) oxidation in LPS stimulated macrophages (as indicated) incubated for 2 h. Fluorescence (excitation 485 nm, emission 520 nm) was measured in Relative Fluorescence Units (RFU); the oxidation rate was expressed as RFU/min. *Panel B*: Macrophages stimulated with 20 ng/ml LPS, were incubated in the presence or absence of 500 μM CEES (as indicated) for 24 h. Post, CEES was applied after the 24 hours of LPS stimulation; Sim, CEES was applied simultaneously with LPS. *Panel C*: LPS stimulated cells were incubated in the presence or absence of 25 μM ebselen, a selective iNOS inhibitor (as indicated). 10, 10 ng/ml LPS; 20, 20 ng/ml LPS. Mean values not sharing a common letter are significantly different (p < 0.05).

Using DCFH-DA and DAF-2DA, we were able to continuously monitor NO formation in intact macrophages under a variety of conditions. Previously, we [18] and others [19] have shown that LPS exclusively induces the iNOS form of nitric oxide synthase in murine macrophages. Figure 3a shows DCFH oxidation in RAW 264.7 cells stimulated with different levels of LPS for 24 hours. In the absence of LPS, the rate of DCFH oxidation was extremely low but increased with increasing exposure to LPS; however, this effect was nearly saturated at LPS levels above 15 ng/ml.

We then measured the rates of DAF-2 oxidation in RAW 264.7 macrophages stimulated with 20 ng/ml LPS in the presence or absence of 500 μM CEES during 24 hour incubations (Figure 3b). In the absence of LPS or CEES, minimal DAF-2 oxidation was observed. As expected, LPS alone induced a marked increase in DAF-2 oxidation. Next, macrophages incubated with LPS for 24 hours were then exposed (post-treatment) to 500 μM CEES and the rate of DAF-2 oxidation immediately measured. As shown in Figure 3b, there was no change in rate of DAF-2 oxidation compared to cells treated with LPS alone. These data strongly support the notion that CEES does not directly inhibit iNOS enzymatic activity. Similar results were obtained with DCFH-DA staining (data not shown). As expected, macrophages simultaneously treated with both LPS and CEES for 24 hours show a marked decrease in either DAF-2 or DCFH oxidation.

To further confirm that DCFH oxidation is overwhelmingly due to iNOS, we incubated LPS-stimulated macrophages with ebselen (see Figure 3c). Ebselen is a selenoorganic compound that can inhibit both the activity of iNOS [20] and its induction by LPS [21]. Ebselen (25 μM) almost completely inhibited the DCFH oxidation in RAW 264.7 cells treated with 10 ng/ml or 20 ng/ml LPS. Ebselen was not cytotoxic at the levels used in Figure 3 (data not shown).

## Discussion

Overall, the experiments detailed in this work show that CEES treatment in LPS-stimulated RAW264.7 murine macrophages transiently inhibits intracellular NO generation by interfering with iNOS expression rather than by direct inhibition of iNOS enzymatic activity. CEES (as well as HD) undergo rapid hydrolysis in aqueous solutions and this may account, in part, for the transitory nature of its inhibiting effect on iNOS induction [22]. LPS is a major component of the cell wall of gram-negative bacteria and is known to trigger a variety of inflammatory reactions in macrophages and other cells expressing CD14 receptors [23,24]. LPS is ubiquitous and is present in serum, tap water, and dust. Military and civilian personnel would, indeed, always have some degree of exposure to environmental LPS.

LPS stimulation of macrophages is known to involve the activation of protein phosphorylation by kinases as well as the activation of nuclear transcription factors such as NF-κB [25-28]. An important consequence of NF-κB activation in macrophages is the induction of iNOS expression followed with highly elevated NO production [12]. Nitric oxide has been demonstrated to have an important role in promoting cell death; however, the precise nature of this role varies with cell type and the dose. Low levels of nitric oxide protect RAW 264.7 macrophages from hydrogen peroxide induced apoptosis [29], however, nitric oxide has also been reported to induce apoptosis in J774 macrophages [14]. Nitric oxide can induce cell death through energy depletion-induced necrosis and oxidant-induced apoptosis.

We are currently exploring the potential molecular mechanism(s) whereby CEES interferes with iNOS expression in LPS stimulated macrophages. It is possible that GSH depletion caused by CEES determines iNOS expression. There are strong evidences suggesting that thiol depletion and iNOS expression are interrelated [30-32]. For example, LPS stimulated macrophages depleted of GSH exhibit a decreased level of iNOS protein and nitrite production [32]. Similarly, both *in vitro *[30] and *in vivo *[31] studies show that hepatocytes depleted of GSH have a diminished production of nitric oxide which is primarily due to a decreased level of iNOS mRNA. Vos et al. [31] have also presented evidence showing that GSH modulation of iNOS expression in hepatocytes is correlated with NF-kB activation, i.e., GSH depletion is associated with a lack of NF-kB activation. The influence of GSH depletion is not, however, consistent in all cell types. Glucose induced reduction of GSH in intestinal epithelial cells is associated with NF-kB activation and upregulation of iNOS gene expression [33].

It is also possible that CEES decreases iNOS expression by interfering with the LPS-induced activation of transcription factor NF-κB and/or signal transducer and activator of transcription-1α (STAT-1α). It is interesting, therefore, that Gray [34] has found that both CEES and HD inhibit the *in vitro *binding of transcription factor activating protein-2 (AP-2) via alkylating the AP-2 DNA consensus binding sequence rather than by direct damage to the AP-2 protein. Furthermore, it is significant that neither CESS nor its hydrolysis products were found to damage the AP-2 transcription factoring in a manner that prevented its DNA binding [35]. Similar experiments have yet to be done with NF-κB. Chen et al. [36] have also found that nitrogen mustard (bis(2-chloroethul) methylamine) similarly inhibits the binding of AP-2 to its consensus sequence. Nitrogen mustard also was shown to inhibit the binding of NF-κB to the GC-rich consensus sequence due to the interactions with DNA [37]. It is possible, therefore, that CEES also alkylates the NF-κB consensus sequence thereby preventing the binding of the NF-κB to the iNOS promoter. LPS and/or cytokine-inducible NF-κB binding elements of the murine iNOS promoter have been identified [38], and they are rich of guanine, which is the major alkylation site for HD or CEES. The possible effect of CEES on iNOS promoter regulation is currently being explored.

Although the activation of NF-κB due to mustard or CEES exposure have been shown in various cell lines [7,37,39], the detailed mechanism of this event is still unclear. Recent report [39] showed that NF-κB-driven gene expression has maximum at 9 hours in HD treated keratinocytes. In contrast, in a guinea pig model, Chatterjee et al. [40] have shown that NF-κB activation in lung tissues occurs shortly after CEES expose (1 hour), then disappears within 2 hours completely. However, in our experiments we did not observe any short term stimulating effect of CEES on NO production or iNOS expression (data not shown). Notably, the electrophoretic mobility shift assays used by Chatterjee et al. to measure NF-κB activation show only the state of NF-κB protein complex and provide no information regarding its binding to the DNA consensus sequences.

The physiological significance of potentially decreased iNOS expression by exposure to CEES or HD is not known. Considerable evidence, however, supports the view that nitric oxide production via iNOS plays a key role in wound healing [41-43]. Animal studies [16] have shown that the iNOS knockout mice have impaired wound healing that is reversed by iNOS gene transfer. Soneja et al. [44] have suggested that wound healing could be accelerated under circumstances where oxidative stress is minimized and nitric oxide production enhanced. We have initiated work to explore the role of antioxidants in preventing HD induced pathology in skin.

## Conclusion

Our results show that CEES transiently inhibits NO production in LPS stimulated macrophages by inhibiting the expression of iNOS protein and not by modulating the enzymatic activity of iNOS. The decreased iNOS expression induced by CEES suggests that this alkylating agent inhibits the LPS stimulated activation of NF-κB and/or STAT-1α transcription factors, and this possibility is being investigated. We cannot directly address the physiological significance of our *in vitro *results, however, both decreased expression of iNOS and decreased production of nitric oxide are associated with impaired wound healing [16,41,43,44]. It is likely that the CEES or HD toxicity is modulated by a complex balance between nitric oxide production, thiol depletion and oxidative stress.

## Methods

### Materials

RPMI-1640 medium without phenol red and fetal bovine serum with a low endotoxin level were purchased from Life Technologies (Gaithersburg, MD). Rabbit anti-mouse iNOS antibody was obtained from Transduction Laboratory (Lexington, KY). Horseradish peroxidase conjugated anti-rabbit polyclonal antibodies, Escherichia coli lipopolysaccharide serotype 0111:B4, 3-(4,5-dimethylthiazolyl-2)-2,5-diphenyltetrazolium bromide (MTT), and 2-chloroethyl ethyl sulphide were obtained from Sigma Chemical Company (St. Louis, MO).

### Cell culture and treatments

RAW264.7 murine macrophage-like cells (American Type Culture Collection, Rockville, MD) were cultured at 37°C in a humidified incubator with 5% CO_2 _in RPMI-1640 medium with 10% fetal bovine serum, 50 U/ml penicillin and 50 mg/ml streptomycin (GiBcoBRL Grand Island, NY). CEES was used as a fresh (2 week old or less) 50 mM stock solution in dried ethanol. LPS was prepared as a 1 mg/ml stock solution in PBS and stored at -20°C for up to 3 months.

### MTT assay

The MTT (3-(4,5-dimethylthiazool-2yl)-2,5-diphenyltetrazolium bromide) assay was performed by a slight modification of the method described by Wasserman et al. [45,46]. Briefly, at the end of each experiment, cultured cells in 96 well plates (with 200 μl of medium per well) were incubated with MTT (20 μl of 5 μg/ml per well) at 37°C for 4 hours. The formazan product was solubilized by addition of 100 μl of dimethyl sulfoxide (DMSO) and 100 μl of 10% SDS in 0.01 M HCl and the OD measured at 575 nm (Molecular Devices SPECTRAmax Plus microplate reader).

### Western blot analysis

Cellular protein lysates were prepared as described in the protocol from Transduction Laboratory (Lexington, KY). Briefly, about 10^6 ^adherent cells were rinsed once with cold PBS and solublized by boiling in 0.1 ml of SDS-PAGE sample buffer for 5 min. Protein concentration was determined by the BCA protein assay (Pierce Chemical Co., Rockford, IL). A 30 μg aliquot of protein was separated via 8% SDS-PAGE and electrotransferred onto a nitrocellulose membrane. Western blotting was performed with a rabbit polyclonal antiserum against the C-terminal (961 to 1144 amino acids) sequence of mouse iNOS (Transduction Lab, Lexington, KY). The protein was detected using an enhanced chemiluminescence kit from Amersham Life Science (Arlington Heights, IL). Murine iNOS (Calbiochem, CA) was used as a positive control.

### Determination of NO production

The production of NO, reflecting cellular NO synthase activity, was estimated from the accumulation of nitrite (NO_2_^-^), a stable breakdown product of NO, in the medium. Nitrite was measured using the Griess reagent according to the method of Green et al. [47]. Briefly, an aliquot of cell culture medium was mixed with an equal volume of Greiss reagent which reacts with nitrite to form an azo-product. Absorbance of the reaction product was determined at 532 nm using a microplate reader (Molecular Devices Microplate Reader). Sodium nitrite was used as a standard to calculate nitrite concentrations.

### Intracellular NO measurement

Assays were performed using 96-well tissue culture plates as described by Imrich and Kobzik [17]. The cell density was adjusted to 2 × 10^5^/ml, and a 100 μl aliquot of the cell suspension in media was placed put in each well. CEES and LPS solutions to achieve desired concentrations were added and the plate incubated for 24 h at 37°C in 5% CO_2_. Following the removal of media, serum free 1640 RPMI supplemented with 10 mM HEPES containing 20 μM DCFH-DA or 10 μM DAF-2DA (final concentration) was added, and the plates incubated for 2 h at 37°C. Fluorescence intensity (relative fluorescence unit, RFU) was continuously monitored using 485 nm for excitation and 520 nm emission in a florescence microplate reader (FluoStar Microplate Reader, BMG).

### Statistical analyses

Data were analyzed by followed with the Scheffe test for significance with p < 0.05. Results were expressed as the mean ± SD. In all the Figures, mean values not sharing a common letter are significantly different (p < 0.05). Mean values sharing a common letter are not significantly different. The mean values and standard deviations of at least three independent experiments are provided in all the Figures.

## Abbreviations

HD, sulphur mustard gas

CEES, 2-chloroethyl ethyl sulphide

LPS, lipopolysaccharide

NO, nitric oxide

iNOS, inducible nitric oxide synthase

NF-κB, nuclear factor kappa B

STAT-1α, signal transducer and activator of transcription-1α

DCF, dichlorofluorescein

DCFH, dichlorofluorescin

DCFH-DA, dichlorofluorescin diacetate

TNF-α, tumor necrosis factor-alpha

IL-1β, interleukin-1 beta

AP2, activating protein 2

MTT, 3-(4,5-dimethylthiazool-2yl)-2,5-diphenyltetrazolium bromide

DMSO, dimethyl sulfoxide

DEM, diethylmaleate

BSO, buthionine sulfoximine 

DAF-2DA, 4,5-diaminofluorescein diacetate

## Authors' contributions

WLS supervised the overall conduct of the research, which was performed in his laboratory. MQ and HY carried out all of the experimental work in this study and performed the statistical analyses. WLS and VP analyzed the data and drafted the manuscript. MS (along with WLS) conceived of the study, participated in the study design, and provided continuous evaluation of the experimental data. All authors read and approved the final manuscript.
